# Inflammation, Cognition, and White Matter in Older Adults: An Examination by Race

**DOI:** 10.3389/fnagi.2020.553998

**Published:** 2020-10-30

**Authors:** Elizabeth A. Boots, Karla J. Castellanos, Liang Zhan, Lisa L. Barnes, Lisa Tussing-Humphreys, Sean C. L. Deoni, Melissa Lamar

**Affiliations:** ^1^Department of Psychology, University of Illinois at Chicago, Chicago, IL, United States; ^2^Rush Alzheimer’s Disease Center, Rush University Medical Center, Chicago, IL, United States; ^3^Institute for Health Research and Policy, University of Illinois at Chicago, Chicago, IL, United States; ^4^Department of Electrical and Computer Engineering, University of Pittsburgh, Pittsburgh, PA, United States; ^5^Department of Psychiatry and Behavioral Sciences, Rush University Medical Center, Chicago, IL, United States; ^6^Division of Academic Internal Medicine and Geriatrics, College of Medicine, University of Illinois at Chicago, Chicago, IL, United States; ^7^University of Illinois Cancer Center, Chicago, IL, United States; ^8^Advanced Baby Imaging Lab, Women and Infants Hospital, and Department of Pediatrics, Warren Alpert Medical School of Brown University, Providence, RI, United States

**Keywords:** neuroimaging, executive function, inflammatory markers, myelin, racial differences

## Abstract

**Objectives:**

Non-Latino Black adults have greater risk for Alzheimer’s dementia compared to non-Latino White adults, possibly due to factors disproportionally affecting Black adults including cardiovascular disease (CVD). Chronic peripheral inflammation is implicated in both Alzheimer’s dementia and CVD and is known to impact cognition and cerebral white matter, yet little work has examined these associations by race. This study examined associations between inflammation, cognition, and cerebral white matter generally, and by race.

**Methods:**

Eighty-six non-demented older Black and White participants (age = 69.03; 50% female; 45% Black participants) underwent fasting venipuncture, cognitive testing, and MRI. Serum was assayed for interleukin-6 (IL-6), C-reactive protein (CRP), and interleukin 1-beta. Cognitive domains included memory, executive function, and attention/information processing. MRI measures included white matter hyperintensity volumes (WMH) and quantification of white matter integrity in areas outside WMHs via DTI-derived fractional anisotropy (FA) and mean diffusivity, as well as multi-component relaxometry derived myelin water fraction (MWF).

**Results:**

Black and White participants did not differ on age, sex, or CVD risk. Separate linear regression models adjusting for relevant confounders revealed that higher IL-6 associated with lower executive function and higher CRP levels associated with lower FA and MWF. Stratified analyses revealed that these association were significant for Black participants only.

**Discussion:**

These findings suggest that peripheral inflammation is inversely associated with select cognitive domains and white matter integrity (but not WMHs), particularly in older Black adults. It is important to consider race when investigating inflammatory associates of brain and behavior.

## Introduction

Given the number of unsuccessful pharmacological trials in Alzheimer’s dementia (Alzheimer’s Association, 2019), researchers have turned toward alternative approaches to prevent Alzheimer’s dementia prior to its onset, including identification of modifiable risk factors that have established interventions, such as cardiovascular disease risk factors (CVD-RFs). Several CVD-RFs, including hypertension, diabetes, and obesity, have been linked to cognitive decline and Alzheimer’s dementia (DeRight et al., 2015); however, the underlying mechanisms driving these associations have not been fully elucidated. This is particularly true for non-Latino Black adults, who are historically underrepresented in scientific research despite disparities in Alzheimer’s dementia and CVD-RFs. In fact, disproportionately higher levels of uncontrolled CVD-RFs in Black adults (Barnes and Bennett, 2014; Howard et al., 2017) are hypothesized to contribute to their greater risk for Alzheimer’s dementia compared to non-Latino White adults (Mayeda et al., 2016). Therefore, understanding the underlying mechanisms that may explain the relationships of these risk factors to cognition in normal and pathological aging both across and within race is an urgent public health need.

One potential underlying mechanism to explain differential risk for CVD-RFs and their impact on Alzheimer’s dementia in older Black adults is the known chronically elevated levels of peripheral inflammation in Black adults compared with White adults (Nazmi and Victora, 2007). Peripheral inflammatory markers are elevated in aforementioned conditions more common in older Black adults including CVD-RFs (Golia et al., 2014; Bourassa and Sbarra, 2017) and Alzheimer’s dementia (Perry, 2004); however, the directionality of inflammation impacting CVD-RFs or vice versa remains unclear. Peripheral inflammatory markers are thought to communicate with the brain, specifically microglia, via several pathways including active transport of inflammatory cytokines across the blood-brain barrier (BBB), signaling across intact BBB, and circumventricular organs (Perry, 2004; Holmes, 2013). In turn, microglia become activated and more prone to release pro-inflammatory cytokines in response to “foreign” matter such as β-amyloid (Heneka et al., 2015; Bourassa and Sbarra, 2017). With rising β-amyloid accumulation, microglia become highly sensitive to activation, aggravate the β-amyloid clearing process, and chronic central inflammation ensues. This may serve as a catalyst for cerebral white matter damage (Raj et al., 2017) and cognitive decline that may lead to dementia (Perry, 2004; Heneka et al., 2015). In fact, prior studies in older White adults show direct associations between elevated peripheral inflammation and dementia (Holmes et al., 2009; Swardfager et al., 2010) as well as alterations in cerebral white matter and cognition (Wersching et al., 2010; Jefferson et al., 2011; Bettcher et al., 2012; Satizabal et al., 2012; Tegeler et al., 2016). There are few studies examining these associations in older Black adults.

To date, we are aware of nine studies investigating peripheral inflammation and brain aging that have included non-Latino Black participants as greater than 25% of the study sample. Three studies did not expressly examine race; however, they did report associations between higher C-reactive protein (CRP) and (1) lower memory and visuospatial function (Noble et al., 2010), (2) greater white matter damage in the form of white matter hyperintensities (WMH; Raz et al., 2012), and (3) lower white matter integrity measured by diffusion tensor imaging (DTI; Bettcher et al., 2015). These results are similar to those reported for predominantly White cohorts (Wersching et al., 2010; Jefferson et al., 2011; Bettcher et al., 2012; Satizabal et al., 2012; Tegeler et al., 2016). Of the six studies that explicitly investigated race, two cross-sectional studies reported that higher inflammation, measured by CRP, interleukin-6 (IL-6), and interleukin-8 (IL-8) (Windham et al., 2014; Goldstein et al., 2015), was associated with lower cognition in older Black adults more so than older White adults. Two longitudinal studies showed no racial differences on the effect of inflammation on cognitive change (Yaffe et al., 2003; Beydoun et al., 2018). One longitudinal study found that greater inflammation in midlife predicted poorer white matter integrity, i.e., greater mean diffusivity (MD), two decades later in Black adults more so than White adults (Walker et al., 2017); however, this same group did not show racial differences between change in inflammation from middle to late-life and late-life WMH or DTI-derived white matter integrity (FA; Walker et al., 2018). While these studies and others with smaller percentages of Black participants in the study sample (Zahodne et al., 2018) contribute to the much-needed literature on the relationships between inflammation and brain aging in Black adults, results are mixed, and newer neuroimaging techniques may further enhance our understanding of cerebral white matter associates.

The aims of the current study are two-fold. One, we will examine whether markers of inflammation, including CRP, IL-6, and IL1-beta (IL1-β), are associated with global cognitive function and cognitive domains of memory, executive function, and attention/information processing in a cohort of older Black and White adults, both generally and by race. We hypothesize that higher levels of inflammation will be associated with worse cognition globally and in memory, executive function, and attention/information processing speed, and that these associations will be present in Black participants more so than White participants. Two, we will determine whether CRP, IL-6, and IL1-β are associated with cerebral white matter, generally and by race, using a series of neuroimaging techniques to assess white matter damage (WMH volumes) and white matter integrity, including DTI measures of fractional anisotropy (FA) and MD, and a newer metric of underlying white matter microstructure – myelin water fraction (MWF) derived from multi-component driven equilibrium single pulse observation of T1/T2 (mcDESPOT). We hypothesize that higher levels of inflammation will be associated with higher WMH volumes, lower FA, higher MD, and lower MWF, particularly in Black participants when compared with White participants.

## Materials and Methods

### Participants

This is a secondary analysis of data from a study of vascular brain aging conducted at the University of Illinois at Chicago (UIC) Department of Psychiatry. The study was approved by the UIC Institutional Review Board (IRB) and conducted in accordance with the Declaration of Helsinki with written informed consent obtained from all participants. All requisite IRB and institutional data use agreements were in place prior to data analysis at the Rush Alzheimer’s Disease Center.

As outlined previously (Boots et al., 2019; Bronas et al., 2019), adults aged 60 years or older living in the Chicagoland area were recruited via community outreach. Interested individuals underwent telephone screening to determine initial study eligibility. At this screen, exclusion criteria consisted of self-reported current/past history of neurological conditions including Alzheimer’s or other dementia, mild cognitive impairment, Parkinson’s disease or other movement disorders, stroke, epilepsy, or Axis I or II disorders, a history of head injury or loss of consciousness, a present/past history of substance abuse or dependence, current psychotropic medication use, or contraindications for magnetic resonance imaging (MRI). History of stable (e.g., diabetes) or remitted medical illness (e.g., cancer) was not an exclusionary factor. Individuals were not eligible if they received cognitive testing in the year prior to their participation in this study.

Individuals who passed this telephone screen underwent a more intensive in-person evaluation of inclusion/exclusion criteria. This secondary screening consisted of affective and cognitive screens including the Structured Clinical Interview for DSM-IV-TR (SCID) and the Mini Mental State Examination (MMSE) conducted by a trained research assistant and followed by an evaluation by a psychiatrist, blind to all screening information, who administered the 17-item Hamilton Depression Rating Scale (HAM-D). Final inclusion criteria consisted of an absence of psychiatric symptoms based on the SCID, a score ≤8 on the HAM-D, a MMSE score ≥24, and absence of subjective memory complaints.

The overall study sample consisted of 121 individuals. Given that the current analyses focused on associations of inflammatory markers with cognition and cerebral white matter in non-Latino White and Black adults, we excluded 18 individuals who self-identified as from a Latinx background. Additionally, we excluded 17 participants that did not have at least two of the three inflammatory markers used in this study. Thus, the final analytic sample consisted of 86 non-Latino Black (*n* = 39) and White (*n* = 47) participants.

### Study Protocol

#### Inflammatory Markers

Participants completed a 12-h fasting blood draw in the morning with a registered nurse in the Center for Clinical and Translational Science’s Clinical Research Center (CRC) at UIC. Each participant’s 40-ml venous fasting blood sample was processed for serum via centrifugation and stored at −80°C until analyzed. CRP, IL-6, and IL1-β were quantified using blood serum in all but one participant where blood plasma was used in the absence of blood serum. CRP concentrations were measured in duplicate (*n* = 34) and singly (*n* = 68) by quantitative sandwich ELISA (R&D Systems, Minneapolis, MN, United States). The mean intra-assay CV for the duplicate samples was 4.5%. High sensitivity IL-6 concentrations were measured in duplicate by solid-phase enzyme linked immunoassay ELISA. The mean intra-assay coefficient of variability (CV) was 5.5%. The IL-6 variable was log-transformed to normalize its distribution for analyses. IL1-β concentrations were measured by quantitative sandwich ELISA. Due to manufacturer error in the original kits, IL1-β was re-assayed in a restricted sample (*n* = 74) and not performed in duplicate. The 12 participants missing IL1-β did not differ from the 74 participants with IL1-β (data not shown).

#### Cognition

Participants underwent a comprehensive neuropsychological evaluation administered by trained research assistants supervised by a licensed clinical neuropsychologist (ML). As detailed elsewhere (Boots et al., 2019), Principal Component Analysis (PCA) with Varimax Rotation was utilized to statistically group select cognitive test measures with factor loadings >0.6 per rotated component, resulting in the following composite cognitive domains: *Executive Function* – Trail Making Test B minus A, Letter Fluency, Letter Number Sequencing, and Matrix Reasoning*; Attention and Information Processing* – Trail Making Test A, Trail Making Test Motor, and Digit Symbol Coding; *Memory* – California Verbal Learning Test–II Total Learning Trials 1–5, Delayed Free Recall, and Recognition Discriminability. Computation of each cognitive domain z-score involved recoding relevant test items (i.e., multiplied by −1) so higher scores equaled better performance, computing z-scores for each cognitive test item for the analytic sample, then averaging z-scores within an individual cognitive domain for every participant. Global cognition was calculated by averaging the z-scores of all neuropsychological test items outlined above.

#### Neuroimaging Acquisition

Participants underwent neuroimaging at UIC’s Center for Magnetic Resonance Research. Whole brain images aligned to the AC-PC line were acquired on a GE MR750 Discovery 3T scanner (General Electric Health Care, Waukesha, WI, United States) using an 8-channel head coil. Participants were positioned supine on the scanner table with foam pads to minimize head movement and instructed to remain still throughout scanning. T1-weighted images were acquired via Brain Volume (BRAVO) imaging sequence (field of view: FOV = 22 mm^2^; voxel size = 0.42 mm × 0.42 mm × 1.5 mm; 120 interleaved axial slices; TR/TE = 1200 ms/5.3 ms; flip angle = 13°). Multi-slice T2-weighted fluid-attenuated inversion recovery (FLAIR) images were acquired using a two-dimensional PROPELLER sequence (FOV = 22 cm, voxel size = 0.35 mm × 0.35 mm × 3.0 mm, 40 contiguous axial slices, TR/TI/TE = 9500 ms/2500 ms/93.3 ms, flip angle = 142.35°). Diffusion MRI images were axially acquired using 2-D spin-echo EPI sequence [FOV = 20 mm; voxel size = 0.78 mm × 0.78 mm × 3.0 mm; TR/TE = 5525/93.5 ms; flip angle = 90°; 40 interleaved slices in 32 gradient directions, *b* = 1400 s/mm^2^ and 6 baseline (b_0_) images]. mcDESPOT comprised of a series of spoiled gradient echo sequence (SPGR) and fast imaging employing steady-state acquisition (FIESTA) images acquired over a range of flip angles: SPGR: TE/TR = 2.5 ms/5.3 ms, flip angles(α) = [3,4,5,6,7,9,12,17°], receiver bandwidth (BW) = 27.78. FIESTA: TE/TR = 1.6 ms/4.2 ms, α = [12,16,21,27,33,40,51,68°], BW = 41.67 kHz. An inversion recovery SPGR image was acquired to allow correction for transmit magnetic field (B_1_) inhomogeneities; the steady-state free precession (SSFP) data was acquired with two phase-cycling patterns to permit correction for main magnetic field (B_0_) off-resonance effects.

#### Neuroimaging Processing

##### White matter hyperintensities

To quantify white matter damage in the form of WMHs, we followed previously published procedures (Bronas et al., 2019) that involved registering each participant’s T1-weighted BRAVO to their T2-weighted FLAIR using affine registration (FLIRT, FMRIB, University of Oxford, United Kingdom). Brains were extracted from the co-registered images (BET, FMRIB, University of Oxford, United Kingdom). WMHs were automatically segmented using a support vector machine classifier considering both BRAVO and FLAIR information for each participant (WMLS, SBIA, University of Pennsylvania). This measure was log-transformed. Additionally, each participant’s WMH volume was subtracted from their total segmented white matter volume to produce a mask of normal appearing white matter (NAWM) for use as outlined below.

##### Diffusion tensor imaging

Diffusion-weighted images were realigned to the b_0_ image using the automatic image registration algorithm with affine transformation to minimize eddy current distortions. Diffusion tensor elements were computed at each voxel with signals from the 32 diffusion weighted images fitted to obtain the six elements of the diffusion tensor, which were then diagonalized to determine three eigenvalues (λ1, λ2, λ3) and three eigenvectors (v_1_, v_2_, v_3_) using FMRIB Software library (FSL)^1^. Eigenvalue images were used to compute FA and MD maps for each participant. These images were transformed to standard space followed by non-linear warping to normalize images (isotropic 1 mm^3^ voxels) to the Montreal Neurological Institute standard space (MNI152) and then skull and dura stripped (Brain Extraction Tool^2^; Smith, 2002). Images were visually inspected to assure no gross errors occurred during normalization. To quantify white matter integrity via DTI, the extraction of FA and MD data were only completed within NAWM as extracting from whole brain (i.e., including areas with WMHs) could lead to bias in measurement and limit understanding of subtle differences not due to overt white matter damage. Extracted FA and MD values within NAWM were divided by the number of NAWM voxels for each participant to standardize measurement by accounting for differences in NAWM volumes.

##### mcDESPOT

Following image alignment, non-brain signal removal, and correction for main and transmit magnetic field (B_0_ and B_1_) inhomogeneities (Deoni, 2011), a four-pool signal model comprised of myelin-associated water, intra-extra axonal water, cerebrospinal fluid, and a non-exchanging free-water pool was fit to mcDESPOT data to derive voxel-wise myelin-associated water pool maps (Deoni et al., 2013). The four-pool signal model was fit using a stochastic region contraction approach (Deoni et al., 2012). Each participant’s map was non-linearly aligned to a common template using Advanced Normalization Tools (Avants et al., 2014)^3^. To quantify a standardized measurement of white matter integrity via mcDESPOT, these maps were used for extraction of mean MWF content in NAWM and divided by the number of NAWM voxels to account for differences in NAWM.

#### Neuroimaging Quality Control

Of the 86 participants with cognitive data and at least two of three inflammatory markers, four did not complete neuroimaging and two had incidental findings. Of the remaining 80 participants with neuroimaging data, two participants failed quality assurance procedures (1 T1-BRAVO, 1 DTI), and DTI and mcDESPOT data were not available for seven participants at the time of our analyses. In order to maximize sample sizes, we allowed the resulting analytic samples, and ensuing degrees of freedom, to fluctuate based on the analysis being performed (e.g., 79 participants for WMH, 71 for DTI, and 72 for MWF analyses).

#### Covariates

In addition to demographic variables (i.e., age, sex, race), predicted verbal IQ (pVIQ) derived from word reading and the Framingham Stroke Risk Profile (FSRP) were additional *a priori* covariates in all analyses; intracranial volume (ICV) was used as a covariate in MRI analyses. Specifically, pVIQ from the Wechsler Test of Adult Reading was used as a proxy for educational quality, a more informative means of assessing educational attainment than years of education in racially/ethnically diverse samples (Carvalho et al., 2015). Given the relationship between CVD-RFs and peripheral inflammation (Golia et al., 2014), we wanted to ensure models were detecting as specific an effect as possible of inflammation on our outcome variables that was not attributable to CVD-RFs. Thus, we utilized the FSRP 10-year risk of stroke that incorporates age, sex, systolic blood pressure, anti-hypertensive therapy, diabetes, smoking, cardiovascular disease, and atrial fibrillation to measure CVD-RF burden (Dufouil et al., 2017). Data for the FSRP-10 was obtained via medical history, physical examination, two seated blood pressure readings separated by 5 min, and an electrocardiogram conducted at the UIC CRC. Resulting FSRP-10 scores were log-transformed to normalize the distribution. ICV was derived from each participant’s T1-weighted BRAVO using FreeSurfer 6.0^4^ to account for individual differences in brain size for all neuroimaging analyses.

### Statistical Analyses

Analyses were conducted in SPSS Version 25 with *p* ≤ 0.05 for significance. Due to outliers, IL1-β measures falling three standard deviations from the interquartile range were winsorized. Bivariate Pearson (continuous) or point-biserial (dichotomous) correlations explored unadjusted relationships between predictors, outcomes, and *a priori* covariates.

Multivariate linear regression models evaluated the associations between inflammation (predictor variables CRP, IL-6, and IL1-β, separately) and global cognition (outcome variable) adjusting for age, sex, race, pVIQ, and FSRP-10. To reduce the number of cognitive comparisons, only where global cognitive models were significant did we conduct separate follow-up analyses for specific cognitive domains of executive function, attention/information processing, and memory. Multivariate linear regression models evaluated the associations between inflammation (predictor variables CRP, IL-6, and IL1-β, separately) and outcome variables of white matter damage (WMH) and white matter integrity (FA, MD, and MWF extracted from NAWM, separately). In addition to the covariates noted above, ICV was additionally included in MRI models. All neuroimaging results were corrected for multiple comparisons using false-discovery rate (FDR) correction. Across all analyses conducted in the full sample, where the association of interest was significant, we followed by a model that included a race × inflammatory marker interaction term.

Given our *a priori* hypotheses that significant associations found in the entire sample would be present in Black participants more so than White participants, we also followed up significant full sample results with analyses stratified by race regardless of significance in the interaction models. This decision was bolstered (i) by potential limitations to utilizing interaction terms in health disparities research in the context of racial differences in either predictor or outcome variables (Ward et al., 2019), and (ii) the general need for more thoughtful characterization of data across as well as within racial groups (Whitfield et al., 2008).

## Results

### Participant Characteristics

Participants (50% female) were, on average, 69 years of age, with approximately 15 years of education. All screening measures of cognition and mood were in the normal range, demonstrating the non-demented, non-depressed nature of this sample (Table 1). There were no differences between Black and White participants for age, sex, FSRP-10, cognitive or mood screening measures, or inflammatory markers; however, years of education and pVIQ scores were significantly different between groups (Table 1), as were outcome variables including global cognition and MD – although MD only approached significance at *p* = 0.057.

**TABLE 1:** Sample characteristics, overall and stratified by race.

**Characteristic**	**Overall sample**	**Non-Latino Black participants**	**Non-Latino white participants**	***p*-value**
N	86	39	47	
Age, years	69.03 ± 6.65 *(60–89)*	69.00 ± 6.61 *(60–89)*	69.06 ± 6.75 *(60–85)*	0.965
Female, %	50.0	51.3	48.9	0.831
Degree years of education	15.58 ± 2.67 *(10–22)*	**14.85 ± 2.50 *(10–18)***	**16.19 ± 2.68 *(12–22)***	**0.019**
pVIQ derived from Word Reading	107.98 ± 11.92 *(79–126)*	**99.13 ± 7.92 *(81–110)***	**115.32 ± 9.46 *(79–126)***	**<0.001**
HAM-D total	1.08 ± 1.43 *(0–7)*	1.06 ± 1.75 *(0–7)*	1.09 ± 1.15 *(0–4)*	0.923
MMSE total	28.79 ± 1.35 *(25–30)*	28.59 ± 1.45 *(25–30)*	28.96 ± 1.27 *(25–30)*	0.212
FSRP-10	6.69 ± 5.13 *(1.08–26.28)*	7.09 ± 5.45 *(1.76–26.28)*	6.35 ± 4.87 *(1.08–17.49)*	0.510
IL-6, pg/mL	3.94 ± 2.41 *(0.87–10.33)*	4.37 ± 2.33 *(0.87–9.85)*	3.57 ± 2.45 *(0.91–10.33)*	0.127
CRP, pg/mL	2.97 ± 1.76 *(0.23–6.02)*	3.23 ± 1.84 *(0.23–6.02)*	2.76 ± 1.67 *(0.24–6.02)*	0.216
IL1-β, pg/mL	0.58 ± 0.12 *(0.48–1.45)*	0.56 ± 0.05 *(0.50–0.68)*	0.59 ± 0.15 *(0.49–1.45)*	0.372
Global cognition	0.08 ± 0.62 (−*1.81 to 1.55)*	−**0.16 ± 0.64 (**−***1.81 to 1.55)***	**0.28 ± 0.52 (**−***1.21 to 1.17)***	**0.001**
WMH (log-transformed)	3.33 ± 0.47 *(2.35–4.33)*	3.39 ± 0.41 *(2.42–4.28)*	3.28 ± 0.51 *(2.35–4.33)*	0.322
FA/NAWM Ratio*	2.70 ± 0.16 *(2.32–3.14)*	2.66 ± 0.17 *(2.32–2.97)*	2.72 ± 0.14 *(2.39–3.14)*	0.123
MD/NAWM Ratio*	*0.09 ± 0.03 (0.02–0.16)*	*0.08 ± 0.03 (0.02–0.14)*	*0.10 ± 0.03 (0.04–0.16)*	*0.057*
MWF/NAWM Ratio*	1.01 ± 0.06 *(0.86–1.19)*	1.00 ± 0.06 *(0.86–1.13)*	1.02 ± 0.06 *(0.88–1.19)*	0.193

Values are Mean ± SD (*Range*) unless otherwise stated. *Indicates multiplied by factor of 1,000,000. pVIQ, predicted verbal intelligence quotient derived from Word Reading, i.e., the Wechsler Test of Adult Reading; HAM-D, Hamilton Depression Rating Scale; MMSE, Mini Mental State Examination; FSRP-10, Framingham Stroke Risk Profile 10 year percent risk of stroke; IL-6, Interleukin-6; CRP, C-reactive protein; IL1-β, Interleukin 1-beta; pg/mL, picograms per milliliter; WMH, white matter hyperintensities; FA, fractional anisotropy; MD, mean diffusivity; MWF, myelin water fraction; NAWM, normal appearing white matter. Bolded entries indicate significance at or below a *p*-value of 0.05.

Bivariate correlations between inflammatory markers, global cognitive and neuroimaging outcomes, and *a priori* covariates in the overall sample are shown in Table 2. IL-6 and CRP were negatively correlated with global cognition and select neuroimaging outcomes (*p*-values ≤ 0.042), respectively. IL-6 was negatively correlated with pVIQ (*r* = −0.232; *p* = 0.032). Global cognition significantly correlated with race and pVIQ (*p*-values ≤ 0.001). WMH was positively correlated with age and FSRP-10, while MD significantly correlated with age and ICV albeit in different directions (Table 2). No other correlations were significant. Given between-group differences in pVIQ, we conducted relevant correlations stratified by race. IL-6 was not related to pVIQ for either Black or White participants (*p* ≥ 0.388). Global cognition was positively correlated with pVIQ in Black (*p* = 0.001) but not White (*p* = 0.068) participants.

**TABLE 2:** Unadjusted bivariate correlations between predictors, outcomes, and *a priori* covariates in the overall sample.

	**Global cognition**	**White matter hyperintensities (WMH)**	**Fractional anisotropy (FA)**	**Mean diffusivity (MD)**	**Myelin water fraction (MWF)**
IL-6	−**0.281**	–0.052	–0.044	0.021	–0.021
CRP	–0.064	–0.113	−**0.256**	–0.025	−-**0.239**
IL1-β	0.064	–0.117	0.138	–0.163	0.095
Age	–0.040	−**0.425**	–0.193	−-**0.347**	–0.188
Sex	0.203	–0.022	–0.228	–0.210	–0.066
Race	−-**0.357**	0.112	–0.183	–0.226	–0.154
pVIQ derived from Word Reading	−**0.502**	–0.127	0.084	0.086	0.044
FSRP-10	–0.085	−**0.374**	–0.164	–0.213	–0.171
ICV	−	0.174	0.214	**0.244**	0.049

*Values are Pearson correlation (continuous) or point-biserial correlation (dichotomous) *r*-values. IL-6, Interleukin-6; CRP, C-reactive protein; IL1-β, Interleukin 1-beta; pVIQ, predicted verbal intelligence quotient derived from Word Reading, i.e., the Wechsler Test of Adult Reading; FSRP-10, Framingham Stroke Risk Profile 10 year percent risk of stroke; ICV, Intracranial Volume. Bolded entries indicate significance at or below a *p*-value of 0.05.*

### Inflammatory Markers and Cognition

**FIGURE 1:**
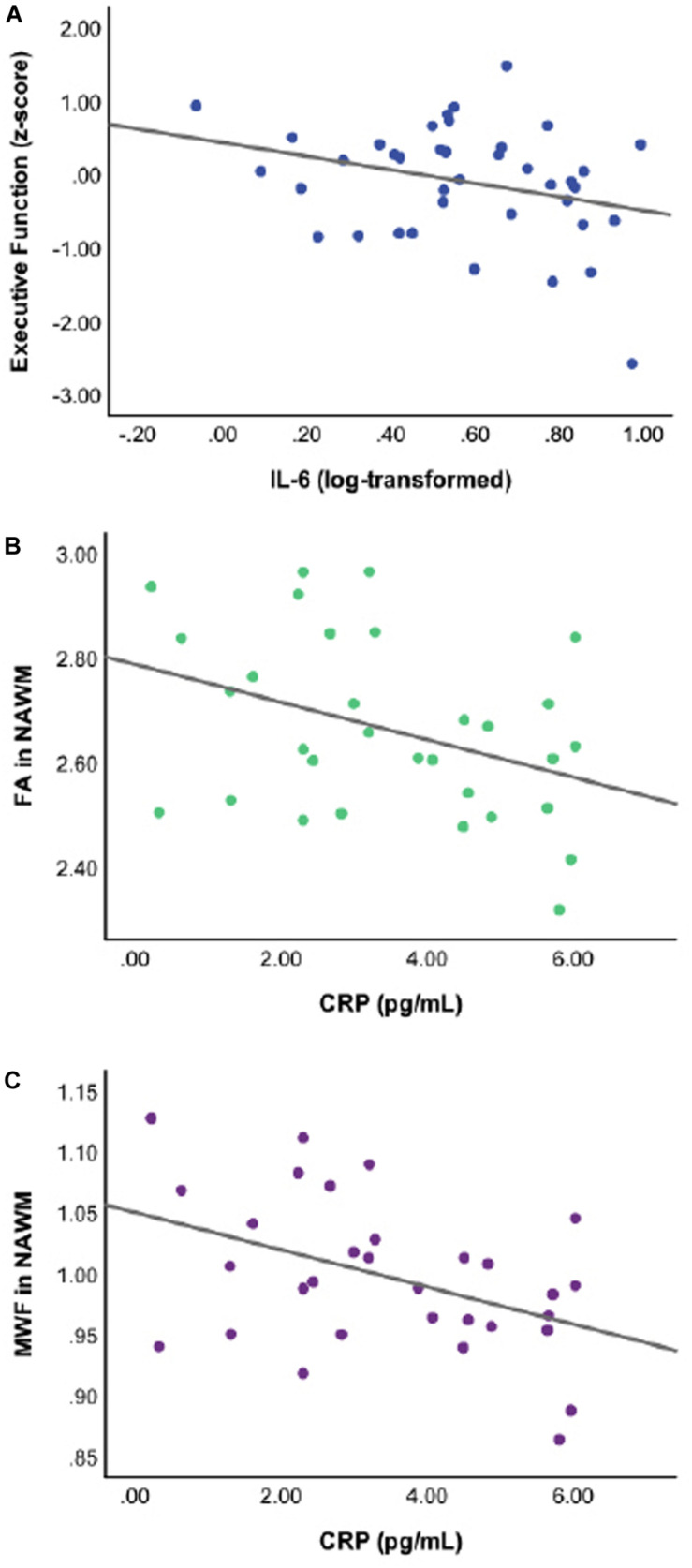
Scatterplots of raw, unadjusted data depicting the significant associations between specific inflammatory markers and cognition or white matter integrity for Black participants at *p* ≤ 0.05. Thus, **(A)** higher levels of interleukin-6 (IL-6) are associated with lower executive function performance; **(B)** higher levels of C-reactive protein (CRP) are associated with lower DTI-derived fractional anisotropy in normal appearing white matter (FA in NAWM); and **(C)** higher levels of CRP are associated with lower myelin water fraction in normal appearing white matter (MWF in NAWM).

Linear regressions adjusted for age, sex, pVIQ, race, and FSRP-10 (Table 3) revealed significant inverse associations between IL-6 and global cognition (*p* = 0.021); no associations were seen between CRP (*p* = 0.694) or IL1-β (*p* = 0.185). Follow-up analyses revealed a significant inverse association between IL-6 and executive function only (*p* = 0.013; Table 3). When we included a race × IL-6 interaction term into our models, there was no significant interaction noted for global cognition or executive function (*p*-values ≥ 0.242). Analyses stratified by race revealed that the association between higher IL-6 and lower executive function was present in Black participants, β(SE) = −0.857 (0.345), *t* = −2.487, *p* = 0.018, η_*p*_^2^ = 0.158 (Figure 1A) but not White participants, β(SE) = −0.475 (0.328), *t* = −1.446, *p* = 0.156, η_*p*_^2^ = 0.049; this same pattern was not revealed in stratified analyses for global cognition (*p*-values ≥ 0.053).

**TABLE 3:** Associations between inflammatory markers and cognition in the overall sample.

**Cognitive measure**	**β (SE)**	***t*-value**	***p*-value**	**partial η ^2^**
***IL-6***				
**Global Cognition**	−**0.486 (0.206)**	−**2.361**	**0.021**	**0.066**
Attention/Information Processing	−0.426 (0.282)	−1.514	0.134	0.028
**Executive Function**	−**0.624 (0.247)**	−**2.530**	**0.013**	**0.075**
Memory	−0.387 (0.333)	−1.160	0.250	0.017
*CRP*				
Global Cognition	−0.022 (0.029)	−0.751	0.455	0.008
*IL1-β*				
Global Cognition	0.080 (0.157)	0.507	0.614	0.004

*IL-6, Interleukin-6; CRP, C-reactive protein; IL1-β, Interleukin 1-beta. Bolded entries indicate significance at or below a *p*-value of 0.05 after adjustment for age, sex, predicted verbal IQ derived from word reading scores from the Wechsler Test of Adult Reading, race, and the Framingham Stroke Risk Profile 10 year percent risk of stroke.*

### Inflammatory Markers and Cerebral White Matter

#### WMH Volumes

There were no significant associations between inflammatory markers and white matter damage as measured by WMH volumes in the full sample (*p*-values ≥ 0.098; Table 4). Given the lack of significance, follow-up interactions and stratified analyses were not conducted.

**TABLE 4:** Associations between inflammatory markers and white matter metrics in the overall sample.

**White matter metric**	**β (SE)**	***t*-value**	***p*-value**	**partial η ^2^**
*IL-6*				
White Matter Hyperintensities	0.012(0.181)	0.066	0.948	< 0.001
Fractional Anisotropy	−0.005(0.072)	–0.063	0.950	< 0.001
Mean Diffusivity	0.006(0.013)	0.455	0.650	0.003
Myelin Water Fraction	−0.003(0.028)	–0.095	0.925	< 0.001
*CRP*				
White Matter Hyperintensities	−0.018(0.029)	–0.643	0.522	0.006
Fractional Anisotropy	−0.022 (0.011)	−1.983	0.052	0.059
Mean Diffusivity	0.0001(0.002)	0.038	0.970	< 0.001
**Myelin Water Fraction**	−**0.009 (0.004)**	−**2.094**	**0.040**	**0.064**
*IL1-β*				
White Matter Hyperintensities	−1.753(1.044)	–1.680	0.098	0.044
Fractional Anisotropy	0.339(0.408)	0.833	0.408	0.011
Mean Diffusivity	0.066(0.071)	0.933	0.354	0.014
Myelin Water Fraction	0.080(0.157)	0.507	0.614	0.004

*IL-6, Interleukin-6; CRP, C-reactive protein; IL1-β, Interleukin 1-beta. Bolded entries indicate significance at or below a *p*-value of 0.05 after adjustment for age, sex, predicted verbal IQ derived from word reading scores from the Wechsler Test of Adult Reading, race, and the Framingham Stroke Risk Profile 10 year percent risk of stroke.*

#### FA and MD in NAWM

C-reactive protein was associated with FA in NAWM in the full sample after adjusting for age, sex, pVIQ, race, FSRP-10, and ICV (*p* = 0.052; Table 4), but did not reach our threshold for significance, regardless of FDR correction for multiple comparisons. No other associations were significant (Table 4). Given the relationship between CRP and FA in NAWM approached significance in the full sample, we included a race × inflammatory marker interaction term to the model; it did not reveal significant effect modification (*p* = 0.166). In contrast, analyses stratified by race revealed that higher levels of CRP were significantly associated with lower FA in NAWM in Black participants, β(SE) = −0.038 (0.014), *t* = −2.722, *p* = 0.012, η_*p*_^2^ = 0.236 (see Figure 1B) but not White participants, β(SE) = −0.005 (0.015), *t* = −0.348, *p* = 0.730, η_*p*_^2^ = 0.004. Stratified analyses survived FDR correction for multiple comparisons.

#### MWF in NAWM

In the full sample, linear regressions adjusted for age, sex, pVIQ, race, FSRP-10, and ICV indicated that higher levels of CRP were also associated with lower white matter integrity in the form of MWF in NAWM (*p* = 0.040; Table 4); however, this result did not survive FDR correction. No other associations were significant (Table 4). Including a race × inflammatory marker interaction term in the model did not reveal significant effect modification (*p* = 0.093). In contrast, analyses stratified by race revealed that higher levels of CRP were associated with lower MWF in NAWM in Black participants, β(SE) = −0.016 (0.005), *t* = −3.070, *p* = 0.005, η_*p*_^2^ = 0.282 (see Figure 1C) but not White participants, β(SE) = −0.001 (0.006), *t* = −0.198, *p* = 0.844, η_*p*_^2^ = 0.001. Stratified analyses survived FDR correction for multiple comparisons.

## Discussion

This cross-sectional study revealed racial distinctions in the associations between peripheral inflammatory markers, cognition, and cerebral white matter in non-demented, non-depressed older adults. Specifically, after adjustment for *a priori* covariates, higher IL-6 was associated with lower global cognition and executive functioning, and higher CRP was associated with lower FA and MWF in NAWM. All associations were driven by Black participants rather than White participants. Together, results point toward differential relationships between inflammatory markers and brain-behavior in older Black and White adults. Our work adds support to the possibility that inflammation may be a risk factor for cognitive decline and more subtle markers of adverse brain health, particularly in Black adults.

The current study adds to the literature in several ways. First, our finding associating IL-6 with global cognition and executive functioning in a racially diverse sample extends prior results historically seen in majority White cohorts (Marsland et al., 2006; Mooijaart et al., 2013; Tegeler et al., 2016) to indicate that inflammation is associated with cognition in both White participants and Black participants. Second, although there were no statistically significant differences across race, the fact that these associations, when stratified by race, were driven by Black participants supports prior cross-sectional reports that older Black adults with higher levels of IL-6 exhibited poorer executive function when compared with older White adults (Windham et al., 2014). Lastly, the lack of significant results for other cognitive domains is similar to previous reports of IL-6 associating with executive function but not memory or attention in some (Mooijaart et al., 2013; Windham et al., 2014), but not all studies involving older Black adults (Jordanova et al., 2007). While differences in defining cognitive domains may help explain conflicting results, e.g., executive function and attention/information processing often include similar neuropsychological tests across studies (Chan et al., 2008), it is also possible that inflammation may impact executive function more so than memory or attention. More work is needed to support the findings in our and other studies (Mooijaart et al., 2013; Windham et al., 2014).

Our study also provided new evidence regarding the relationship between inflammatory markers and white matter integrity across, as well as within race. The inverse relationship between CRP and FA in NAWM in the current study’s diverse sample is consistent with some – but not all (Gu et al., 2017) – cross-sectional (Wersching et al., 2010) and longitudinal (Bettcher et al., 2015; Walker et al., 2018) studies of majority White cohorts. Furthermore, our work expands upon the only other study to date reporting racial differences in CRP and white matter integrity, i.e., DTI-derived MD (Walker et al., 2017) to include an examination of underlying white matter microstructural integrity (i.e., MWF in NAWM). While our results suggest that both White and Black participants have lower MD as well as MWF with higher inflammation, these full sample findings did not survive correction for multiple comparisons and should be interpreted with caution. Despite non-significant interactions by race on these same variables, stratified analyses suggest that higher levels of CRP, particularly in older Black adults more so than White older adults, are also associated with lower levels of white matter integrity, more specifically, myelin integrity. Together with the fact that we and some (Wersching et al., 2010) but not all (Satizabal et al., 2012; Walker et al., 2018) investigators have failed to find a relationship between CRP and WMH suggests that gross metrics like WMH may be too far along in the neurodegenerative process to show an association with inflammation. In contrast, the association between inflammation and white matter integrity, especially myelin integrity in NAWM, may signal a relationship of vulnerability to or milder tissue changes of overt white matter damage as previously shown in head-to-head neuroimaging to neuropathological comparisons within NAWM and WMH (Bronge et al., 2002; Simpson et al., 2007). Longitudinal work is needed to fully elucidate the role of inflammation in the chronicity and/or severity of white matter degeneration across brain tissue, especially in older Black adults.

While relationships between IL-6 and cognition, and separately, CRP and cerebral white matter integrity were observed, no inflammatory marker showed associations with *both* cognition and cerebral white matter. It has been suggested that peripheral IL-6 may be more impactful on cognitive function, executive function specifically, than CRP (Mooijaart et al., 2011, 2013), which is consistent with our findings. Additionally, while IL-6 and CRP are mechanistically linked in the pro-inflammatory response, these inflammatory markers also have distinct signaling pathways (McFadyen et al., 2018). For example, IL-6 engages microglia and upregulates the production of β-amyloid (Wyss-Coray and Rogers, 2012), while CRP targets not only β-amyloid but also brain vasculature (McFadyen et al., 2018). Thus, CRP has an active role in the pro-inflammatory response to cerebrovascular disease including ischemia and atherosclerosis (McFadyen et al., 2018), diseases that negatively impact white matter integrity and underlying white matter microstructure (Papma et al., 2014). Together, these studies provide potential explanations for our results linking IL-6 with cognition and CRP with white matter integrity and demyelination in NAWM; however, there is limited work to explain why these patterns were specific to Black adults.

From a clinical perspective, CVD-RFs and psychosocial stressors have long been hypothesized to contribute to racial disparities in brain aging (Barnes and Bennett, 2014). We tested our hypotheses with and without (data not shown) CVD-RF burden as a covariate; CVD-RF burden as measured by the FSRP-10 did not alter our results. It is possible, however, that related CVD-RFs not accounted for by the FSRP-10, such as obesity and hypercholesterolemia, are playing a role, especially given their greater likelihood in Black adults (Barnes and Bennett, 2014; Howard et al., 2017). Psychosocial stressors, such as lower educational quality (Liu et al., 2014), institutionalized racism and experiences of discrimination (Pascoe and Smart Richman, 2009), and financial and/or socioeconomic fragility (Shaked et al., 2019) may also play important roles in associations between inflammation, cognition, and cerebral white matter. We adjusted for pVIQ as a proxy for educational quality, given significant racial differences in years of education in our sample. While educational quality was associated with select inflammatory markers and global cognitive functioning, it did not account for observed racial differences, suggesting that factors beyond educational quality may be important to consider. For example, higher CRP has been associated with higher levels of self-reported discrimination in older Black adults (Lewis et al., 2010), and CRP has been shown to moderate the association between experiences of discrimination and cognition in older Black adults (Zahodne et al., 2018). It is possible that the inclusion of other social determinants of health may have yielded different results. Relationships between financial and/or socioeconomic fragility, inflammation and brain health may also be influenced by the intersection of sex and race (Gruenewald et al., 2009) and may be mediated, in part, by select CVD-RFs (Akrivos et al., 2020). Thus, future work is needed incorporating not only additional – and perhaps more targeted – CVD-RFs, but critical psychosocial stressors into a comprehensive longitudinal investigation of inflammatory markers and brain-behavior if we are to fully understand race-related patterns in our and others’ results.

While this study has several strengths, it is not without limitations. The cross-sectional nature of our analyses neither allows for a determination of causality in reported relationships nor a definitive explanation of the mechanisms for these effects. Additionally, the high average years of education of our sample, regardless of race, may limit generalizability to the larger population given that only one third of United States adults 65 and older hold a bachelor’s or higher degree (Ryan and Bauman, 2016). Stratified analyses – originally justified given our *a priori* hypotheses and limitations to interaction analyses in health disparities research (Ward et al., 2019) – should, nonetheless, be interpreted with caution given that there were no between-group differences in inflammatory marker predictor variables and only significant racial differences in two of five outcome variables. Further, null findings with IL1-β may have been due to a more limited sample size for this marker, particularly in Black participants. While standardized blood draws were completed in the morning, we did not ask participants about recent illnesses that are known to impact inflammatory marker levels. Furthermore, we did not ask participants about experiences of discrimination or financial and/or socioeconomic fragility and lacked the sample size to investigate the intersectionality of sex and race in our work. Lastly, while excluding individuals with factors associated with inflammation including depression allowed for stronger, unbiased conclusions regarding inflammation and healthy brain aging, it may have limited the variability seen in our inflammatory markers.

Strengths of this study include our comprehensive assessment of cognition and multi-modal interrogation of cerebral white matter damage and integrity, including a novel neuroimaging marker of myelin water fraction. This marker allowed for the detection of subtle differences in white matter that otherwise may go undetected. Finally, our study contributes to a small, but growing literature attempting to understand the increased risk for Alzheimer’s dementia and associated CVD-RF burden in Black adults.

Our work emphasizes the importance of incorporating race into understanding associations in peripheral inflammation as related to cognition and cerebral white matter in older adults. More specifically, there are detectable associations between IL-6 and cognition as well as CRP and cerebral white matter integrity in a non-demented, non-depressed sample of older adults, particularly Black adults. By incorporating subtle metrics of white matter integrity and microstructure within NAWM, the findings of this study also point toward the utility of sensitive neuroimaging markers, such as mcDESPOT, in at risk but not overtly damaged cerebral white matter. The differential associations between inflammation, cognition, and white matter seen by race in this study, if confirmed and extended by longitudinal study, may provide more nuanced understanding of racial disparities in brain aging and Alzheimer’s dementia and point toward the role of reducing inflammatory biomarker levels as potential clinical targets for increasing health equity in brain aging.

## Data Availability Statement

The datasets presented in this article are not readily available because at the time of data collection we did not obtain participant approval for data sharing beyond the study team and named affiliates. Requests to access the datasets should be directed to Melissa_Lamar@rush.edu.

## Ethics Statement

The studies involving human participants were reviewed and approved by the UIC Institutional Review Board (IRB) and conducted in accordance with the Declaration of Helsinki. All requisite IRB and institutional data use agreements were in place prior to data analysis at the Rush Alzheimer’s Disease Center. The patients/participants provided their written informed consent to participate in this study.

## Author Contributions

EB, LT-H, and ML contributed to conception and design of the study. EB and KC organized the database. EB, LZ, SD, and ML performed the statistical analysis. EB wrote the first draft of the manuscript. LT-H and ML wrote sections of the manuscript. All authors contributed to manuscript revision, read, and approved the submitted version.

## Conflict of Interest

The authors declare that the research was conducted in the absence of any commercial or financial relationships that could be construed as a potential conflict of interest.

## Footnotes

1 http://www.fmrib.ac.uk/fsl

2 http://www.fmrib.ox.ac.uk/fsl/

3 http://stnava.github.io/ANTs/

4 https://surfer.nmr.mgh.harvard.edu/
